# Effectiveness of the mobile Stress Autism Mate Junior application in reducing stress and improving quality of life in adolescents with autism: a pilot study

**DOI:** 10.3389/fpsyt.2024.1469257

**Published:** 2025-01-08

**Authors:** Alvin van Asselt, Kirsten Hoeberichts, Sevda Demirel, Anke Scheeren, Yvette Roke

**Affiliations:** ^1^ Emerhese Flevoland, GGz Centraal, Almere, Netherlands; ^2^ Department of Psychiatry and Neuropsychology, School of Mental Health and Neuroscience, Maastricht University, Maastricht, Netherlands; ^3^ Faculty of Behavioural and Movement Sciences, Vrije Universiteit, Amsterdam, Netherlands; ^4^ Amsterdam Public Health, Vrije Universiteit, Amsterdam, Netherlands

**Keywords:** autism, adolescents, mHealth, stress, coping strategies, quality of life

## Abstract

**Objective:**

Studies indicate that stress levels of autistic adolescents may be particularly high. Therefore, support is needed to help them deal with their stressors. Stress Autism Mate (SAM) Junior, a mobile self-help tool, was designed in co-creation with adolescents with autism to help reduce daily stress levels. The app is based on the SAM app, which was previously shown to be effective in reducing stress in autistic adults. This study aimed to evaluate the effectiveness of the SAM Junior app in reducing perceived stress and maladaptive coping styles, and increasing adaptive coping styles and quality of life in adolescents with autism.

**Methods:**

A total of 24 Dutch adolescents with autism participated in this Single Case Experimental Design study. Sixteen of them (9 girls and 7 boys; *M_age_* = 15.0 years, *SD* = 1.9) completed all research phases. Data were collected at four time points separated by four weeks: Control, pre-test, post-test and follow-up. Linear mixed-effects models were used to analyze the data.

**Results:**

At post-test, use of the SAM Junior app had no significant effects on participants’ perceived stress (B = 0.31; 95% CI [-1.59, 2.22], *p* = .73), adaptive coping (B = -1.38; 95% CI [-5.69, 2.94], *p* = .51), maladaptive coping (B = -0.63; 95% CI [-4.56, 3.30], *p* = .74) and quality of life (B = -4.13; 95% CI [-12.19, 3.94], *p* = .29). These non-significant effects persisted at follow-up.

**Discussion:**

Current preliminary results do not show effectiveness of the SAM Junior app to support adolescents with autism. Using the app as intended, without professional supervision, may have been too complex for this population. Further research is needed to determine the potential effects of the SAM Junior app with more certainty.

## 1 Introduction

Autism^1^ is a typically characterized by two core attributes: (1) Different interaction styles and preferences, and (2) A preference for routines and focused interests (2). These characteristics may be particularly impairing during adolescence (3). Apart from sexual maturation, this life stage places an emphasis on developmental tasks that are commonly more challenging in adolescents with autism^2^, such as achieving independence, adapting to novel environments and building relationships (3). These difficulties also makes them more prone to experience one or multiple adverse life events, such as being bullied (5) or maltreated (6). In addition, autistic adolescents use more maladaptive strategies to manage stressors (*coping styles)*, such as rumination and suppression, than their allistic (non-autistic) peers (7). Taken together, these factors partly explain why the perceived stress levels of autistic adolescents are generally high (8, 9). *Perceived stress* is defined as negative thoughts and feelings that arise when people believe they cannot adequately cope with the demands that are being put on them by daily life (10). While low amounts of stress can be adaptive, heightened and chronic stress of adolescents with autism can crucially hinder their development, leading to outcomes such as autistic burnout (11, 12) and reduced academic achievement (13, 14).

Despite the high levels of perceived stress, autistic adolescents experience major barriers in accessing mental health care (15, 16). Reasons for this include, but are not limited to, high costs of services (17), fear of being stigmatized as a mental health patient (15), as well as a perceived lack of expertise in mental health professionals (15, 18). A lack of professional care for adolescents with autism may significantly dampen their *quality of life* (QoL) (19, 20), meaning that they have a lowered perception on their position in life related to their goals, standards, expectations and concerns (21). While increasing prevention, reducing barriers and improving services are crucial, budgets for mental health care are cut almost globally (22). Therefore, alternative ways to assist this population are urgently needed.

Over the past years, digital interventions for individuals with autism have taken a rise in popularity (23). One of these interventions is mobile health (mHealth): Mobile or digital applications (apps) used in health care (24). The purpose of these apps is to support and/or inform users about their mental health in order to improve their self-management and QoL (25, 26). Given its ease of use and cost-effectiveness, mHealth possesses the ability to reach a large group of adolescents with autism at the same time (27, 28). Nevertheless, the effectiveness of mHealth to reduce stress levels in autistic adolescents is largely unknown (23). To our knowledge, only one qualitative study (29) reported on an app with this purpose. This app, SATORI, aims to lower stress levels through breathing exercises. Eight Mexican adolescents with autism were generally positive on its design principles and believed that it could help them to lower stress and anxiety levels. However, quantitative evidence on the effectiveness of mHealth in adolescents with autism is still lacking.

An effective stress-reducing app for autistic adults is the Stress Autism Mate (SAM) (30, 31). The purpose of this app is to improve stress recognition in adults with autism as well as help them to improve their coping skills. This is done by regularly prompting users to answer questions about their activities and stress-related feelings in the past four hours. Thus, the app follows the principles of Ecological Momentary Intervention (32, 33). In addition, the app offers stress-reducing tips that can help users to lower their stress levels immediately. In a pilot study of 15 adults with autism, Hoeberichts et al. (30) found that four weeks of using the SAM app resulted in a reduction in stress levels and an improvement in QoL. A more recent single case experimental design study in 34 adults with autism also concluded that SAM was able to reduce stress levels, although no changes were found with regard to quality of life (31). However, due to the different life and developmental stages, these results cannot be generalized to adolescents with autism.

In summary, adolescents with autism face considerable barriers in accessing traditional mental health care, highlighting the need for alternatives to support their elevated stress levels. mHealth interventions may be a viable and accessible option, but our understanding of their effectiveness in this population remains limited. Therefore, to address this gap, this study evaluated an adaptation of the SAM app specifically tailored to adolescents: *Stress Autism Mate Junior (SAM Junior)*, More specifically, we aimed to assess the effect of the app on autistic adolescents’ perceived stress, coping styles and QoL after four weeks of use and a four-week follow-up phase. Based on the studies by Hoeberichts et al. (30, 31), we hypothesized that the SAM Junior app decreases perceived stress and maladaptive coping styles of participants while increasing their adaptive coping styles and QoL.

## 2 Materials and methods

### 2.1 Study design

**Figure 1:**
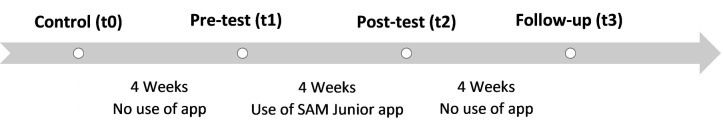
Study design.

This pilot study used a *Single Case Experimental Design (SCED)*. In a SCED all participants provide data multiple times, both before and after an intervention starts, so that they become their own control group (34). In addition, to avoid bias by possible unrelated events (*history bias*), all respondents started at different moments. An ABA-structure was used, meaning that one intervention phase was included (phase B) and surrounded by two phases (A) in which use of the SAM Junior app was prohibited. Data was obtained at four moments separated by four weeks: Control, pre-test, post-test and follow-up (see **Figure 1**). Based on Hoeberichts et al. (30) study on the SAM for adults, it was assumed that this time window would be enough to measure potential effects. Outcomes were perceived stress, adaptive and maladaptive coping styles and QoL.

### 2.2 Participants

Participants were recruited at an outpatient department of two Dutch mental health care facilities, GGz Centraal and Karakter. Both departments provide specialized care for autistic adolescents. Inclusion criteria were (a) a diagnosis of autism according to DSM-V or DSM-V-TR, given by a licensed professional, (b) sufficient intellectual skills and willingness to use the SAM Junior properly, and (c) an age between 12 and 18 years old. Participants were recruited by their mental health professionals as well as through recruitment posters and flyers that were spread throughout the aforementioned organizations. During the course of this study, participants continued to receive care-as-usual.

Based on a power analysis of 1600 Monte Carlo-simulations with a medium effect size of *d* = 0.5, α = .05, and power = .80, 19 participants were needed to reliably answer the research questions. In order to meet this requirement, and to account for potential drop-out, a total of 24 participants were included.

### 2.3 Intervention

SAM was originally developed by GGz Centraal in collaboration with the Dutch organization of applied science research (TNO). Several changes were made to the junior version, such as the addition of age-appropriate activities (e.g. ‘doing homework’, ‘exams’) and stress tips (e.g. ‘watch a movie on your smartphone’), a stress tip appearing immediately upon opening the app, and a longer window of two hours to complete the questionnaires. The changes were based on a co-creation phase with adolescent autistic clients of GGz Centraal. Some personalization options in the SAM Junior app are available (e.g. written text or emoticons in the questionnaire, color schemes and the ability to add personalized activities). No supervision is needed to use the app. The app can be downloaded from the Google Play Store and App Store in 33 countries around the world in seven languages at no cost. More information about the SAM Junior app and the development can be found on https://www.stressautismmate.nl/samjunior/.

#### 2.3.1 Stress Questionnaire in SAM Junior app

In the SAM Junior app, users are prompted to fill in a brief stress questionnaire two to four times a day, depending on personal preference. The user sets the time at which the first questionnaire appears, with subsequent questionnaires appearing at four-hour intervals. Users are given two hours to respond to a questionnaire. If no questionnaire has been filled in within 60 minutes after the first prompt, a reminder will be sent.

The questionnaire starts off with the user selecting one to five activities they have done during the past four hours, and how they felt during them. This list of activities can be personalized. Next are two questions asking the user whether they have felt positive and energized during the past four hours. Seven questions to measure stress levels follow which are based on interviews with adolescents with autism during the testing phase (see **Table 1**). Based on answers of the user, an algorithm calculates the amount of stress during the past four hours (*no stress, a bit of stress, stress* and *much stress*). SAM Junior then asks the user if this score is in sync with their perceived amount of stress, offering the user an opportunity to reflect on their stress levels. Filling in the SAM Junior questionnaire takes two minutes on average.

**Table 1:** Seven main questions of the questionnaire in the SAM Junior application.

Questions
Did you feel irritable?
Does your head feel full?
Were you worried?
Did you have trouble concentrating?
Did you dread activities that are planned in the future?
Did you have negative thoughts?
Did you have anxiety?

Users were asked to answer based on their feelings and thoughts of the past four hours. Answer options were *No; Yes, but not more than normal; Yes, more than normal* and *Yes, much more than normal*. Adding ‘more than normal’ to the answers was advised by autistic adolescents during the co-creation phase of the app in order to help them set their own reference point to answer the questions.

#### 2.3.2 Stress tips

Once the stress score has been calculated, the user is asked whether they wish to receive a stress-reducing tip based on their location (at home or not at home) and level of stress (much stress or lower). This stress tip is randomly based on a preset list that users have selected at the first use of the app. Users were able to change this list later on and could also add their own stress tips. Examples of stress tips are ‘listen to music that helps you relax’ (when at home), ‘go on a walk’ (when not at home) and ‘do a breathing exercise’ (when having much stress).

#### 2.3.3 Weekly overview

**Figure 2:**
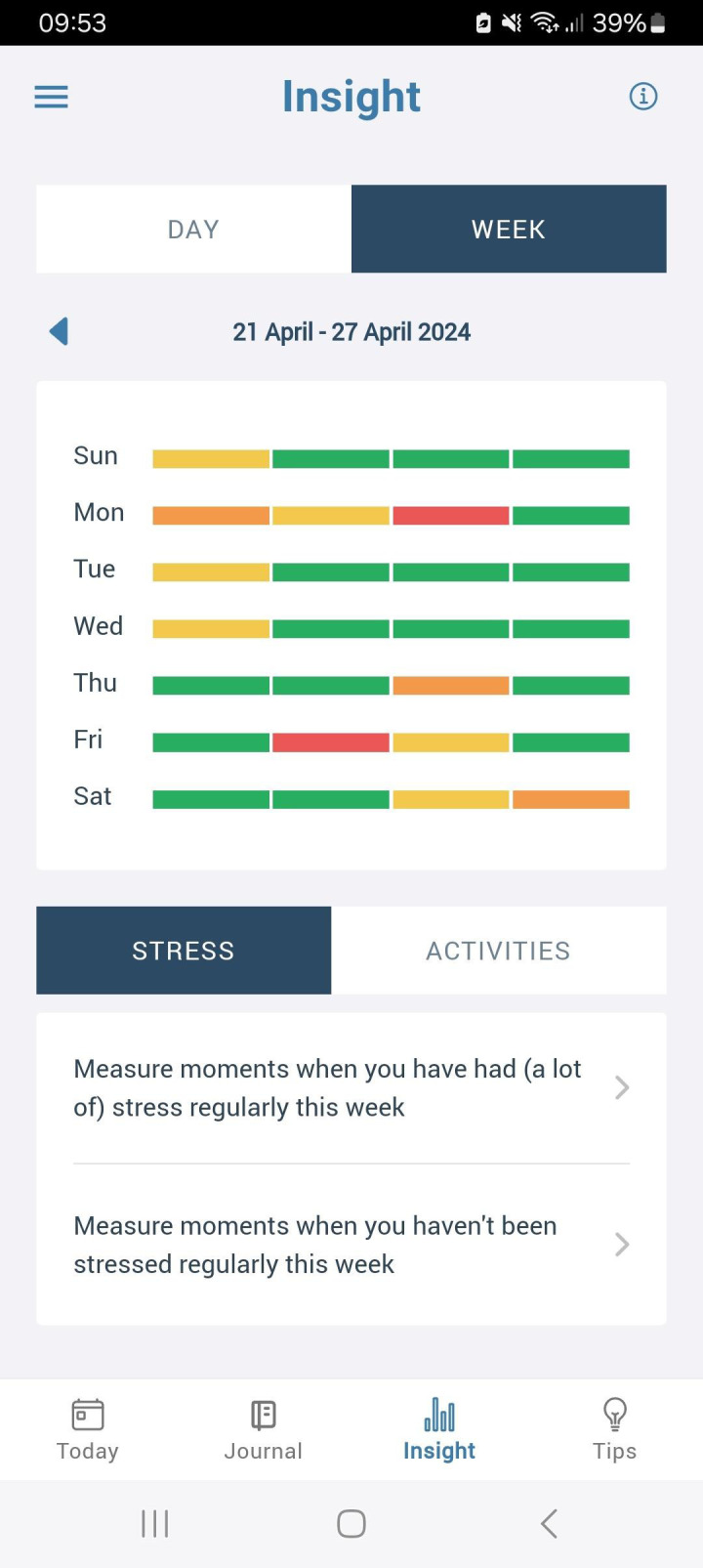
Impression of the interface of the SAM Junior application.

If a user opens the app when no questionnaire is available, a daily and weekly overview is shown of the user’s measured stress scores (see **Figure 2**). Below these charts, times of day and activities that were linked to either little or much stress are highlighted. This overview may aid users to discover patterns in their stress levels. Users are also free to share these overviews with friends, family or practitioners as they desire.

### 2.4 Questionnaires

#### 2.4.1 Perceived stress

Perceived stress scores were obtained with the Dutch version of the *Perceived Stress Scale (PSS)* for adolescents (35, 36). This measure consists of ten items and is commonly used to measure perceived stress in autistic individuals [e.g. (37)]. An example question is ‘*How often did you feel stressed and nervous?*’. Items are scored on a five-point Likert scale ranging from “*Never*” to “*Very often*”. The higher the sum score (range: 0-40), the more perceived stress. Internal consistency of the PSS in this study sample was good (Cronbach’s alpha = .86).

#### 2.4.2 Adaptive and Maladaptive coping

Adaptive and Maladaptive coping styles were measured with the Dutch version of the *Cognitive Emotion Regulation Questionnaire (CERQ)* (38). This 36-item scale measures five adaptive (acceptance, positive refocusing, refocus on planning, positive reappraisal and putting into perspective) and four maladaptive coping styles (self-blame, rumination, catastrophizing, blaming others). Items include ‘*I think of what I can do best*’ for adaptive coping and ‘*I feel that I am the one to blame for it*’ for maladaptive coping. Items are scored on a five-point Likert scale ranging from “*(Almost) Never*” to “*(Almost) Always*”. Although not originally designed for this purpose, scores of adaptive and maladaptive coping styles can reliably be obtained by calculating a total sum score of scores on all respective subscales (39). Therefore, sum scores of adaptive and maladaptive coping styles could range from 20-100 and 16-80, respectively. The scale was found to be reliable in a prior study in adolescents with autism (40). Internal consistency of the CERQ in this study sample was good, with a Cronbach’s alpha of.90 for adaptive and.87 for maladaptive coping styles.

#### 2.4.3 Quality of life

QoL scores were collected with the Dutch version of the *KIDSCREEN-27* (41). This questionnaire consists of 27 items asking the respondent about their feelings and thoughts during the past week. It is often used in studies of autistic adolescents [e.g. (20)]. Five subscales are measured: Physical well-being, Psychological well-being, Autonomy and parent relations, Peers and social support and School environment. An example item is ‘*Has your life been enjoyable?*’. Items were scored on a five-point Likert scale, commonly ranging from “*Not at all*” to “*Always*”. The higher the sum score (range: 27-135), the higher the QoL. Internal consistency of the KIDSCREEN-27 in this study sample was excellent (Cronbach’s alpha = .94).

### 2.5 Procedure

All participants started with a face-to-face or online appointment with a researcher of this study. During this meeting, the first set of questionnaire data was collected through an interview with the researcher. In addition, informed consent and demographic data (age and gender) were obtained. After questionnaire data were obtained during the second (pre-test) appointment, participants installed the SAM Junior app on their mobile phone and were guided through the app and its settings by the researcher. Throughout the intervention phase, a helpdesk was available for all questions and technical issues. During the third (post-test) appointment, participants de-installed the app and were told not to use the app until the follow-up phase had finished. After the participant’s follow-up data were collected, they were free to use the app again.

### 2.6 Data analyses

Linear mixed-effects models were used in SPSS (version 28.0.1.0), which accounts for the fact that effects may differ between individuals (42). Since the participants in this study acted as their own control group, this type of model was suitable. Time of measurement was a fixed (between-subjects) factor, while the participant was a random (within-subject) factor. Participants who did not complete all phases were excluded from the data analysis. Shapiro-Wilk (PSS: *W*(64) = .99, *p* = .91; CERQ-Adaptive: *W*(64) = .99, *p* = .76; CERQ-Maladaptive: *W*(64) = .98, *p* = .33; KIDSCREEN-27: *W*(64) = .97, *p* = .07), Durbin-Watson (PSS: *d* = 1.49; CERQ-Adaptive: *d* = 2.31; CERQ-Maladaptive: *d* = 1.87; kidscreen-27: *d* = 1.53) and visual homoscedasticity tests showed that the data met the assumptions of normality, no significant autocorrelation and homoscedasticity. Therefore, no corrections in order to build the linear mixed-effects models were needed.

For each individual model, it was calculated whether effects occurred on the sum scores of the PSS (perceived stress), CERQ (adaptive and maladaptive coping styles) and KIDSCREEN-27 (quality of life between the pre-test (t1) and post-test (t2) phase, as well as the pre-test (t1) and follow-up (t3) phase. An alpha of.05 was used. Exploratory analyses on the subscales of the CERQ and KIDSCREEN-27 were also conducted. In these analyses, due to the large (28) amount of tests, the Holm-Bonferroni correction for multiple testing was used.

## 3 Results

Of the 24 initial participants, 16 completed all research phases (see **Table 2**). Drop-out mainly occurred due to an inability to reach participants despite multiple attempts and a lack of interest in the app. We did not ask participants to clarify this further. T-tests and a Chi-square test showed no differences between participants who completed the questionnaires at all time points and drop-outs based on age *t*(22) = -.79, *p* = .44, perceived stress on t0, *t*(22) = -.72, *p* = .48, and gender, χ^2^ (1, *N* = 24) = 0.08, *p* = .77. The group of participants who went through all research phases consisted of 9 girls and 7 boys (*M*_age_ = 15.0, *SD* = 1.9), and their average perceived stress at t0 was 29.8 on a scale of 0 to 40, a high score [> 27; (36)].

**Table 2:** Number of participants per research phase.

Time of Measurement	*n*	%
Control (t0)	24	100%
Pre-test (t1)	23	95.8%
Post-test (t2)	17	70.8%
Follow-up (t3)	16	66.7%

### 3.1 Outcomes

#### 3.1.1 Pre-test (t1) to post-test (t2)

The linear mixed-effects models showed no effects of the SAM Junior app from pre-test to post-test (PSS: B = 0.31; 95% CI [-1.59, 2.22], *p* = .73; CERQ-Adaptive: B = -1.38; 95% CI [-5.69, 2.94], *p* = .51; CERQ-Maladaptive: B = -0.63; 95% CI [-4.56, 3.30], *p* = .74; KIDSCREEN-27: B = -4.13; 95% CI [-12.19, 3.94], *p* = .29) (see **Table 3**). Effect sizes were either small or trivial: PSS: *d* = 0.04; CERQ-Adaptive: *d* = -0.11; CERQ-Maladaptive: *d* = -0.07; KIDSCREEN-27: *d* = -0.26.

**Table 3:** Average scores on scales per time of measurement and results of the linear mixed-effects analyses with time of measurement as fixed factor, participants as random factor and perceived stress, adaptive and maladaptive coping styles and QoL as independent variables (N = 16).

Variable	Range	Control (t0)	Pre-test (t1)	Post-test (t2)	Follow-up (t3)	Pre-test (t1) → Post-test (t2)				Pre-test (t1) → Follow-up (t3)			
		*M (SD)*	*M (SD)*	*M (SD)*	*M (SD)*	B	*SE*	*t*	*p*	B	*SE*	*t*	*p*
**Perceived Stress (PSS)**	**0-40**	**29.8 (6.7)**	**29.3 (7.0)**	**29.6 (6.6)**	**29.5 (7.7)**	**0.31**	**0.89**	**0.35**	**.73**	**0.25**	**0.85**	**0.29**	**.77**
**Adaptive Coping Styles (CERQ)**	**20-100**	**56.1 (14.6)**	**52.4 (12.7)**	**51.0 (13.2)**	**53.8 (13.1)**	**-1.38**	**2.03**	**-0.68**	**.51**	**1.44**	**1.81**	**0.79**	**.44**
Acceptance	4-20	11.8 (3.3)	10.9 (2.8)	9.9 (3.2)	10.9 (3.9)	-0.94	0.71	-1.32	.21	0.00	0.66	0.00	1.00
Refocus on Planning	4-20	11.9 (3.9)	10.9 (3.8)	11.2 (4.1)	11.6 (3.3)	0.25	0.62	0.41	.69	0.69	0.55	1.26	.23
Positive Refocusing	4-20	11.1 (3.6)	10.9 (4.1)	9.9 (3.9)	11.4 (4.2)	-1.00	0.87	-1.15	.27	0.44	0.87	0.50	.62
Positive Reappraisal	4-20	10.8 (4.2)	9.4 (4.0)	10.6 (4.3)	10.0 (4.1)	1.19	0.44	2.70	.02*	0.63	0.54	1.16	.26
Putting into Perspective	4-20	10.4 (3.5)	10.3 (3.6)	9.4 (3.1)	9.9 (3.5)	-0.88	0.51	-1.73	.11	-0.31	0.65	-0.48	.64
**Maladaptive Coping Styles (CERQ)**	**16-80**	**37.2 (9.8)**	**34.3 (10.9)**	**33.7 (7.4)**	**35.4 (9.1)**	**-0.63**	**1.84**	**-0.34**	**.74**	**-0.94**	**3.40**	**-0.28**	**.79**
Self-blame	4-20	11.3 (4.2)	9.9 (4.5)	9.9 (3.5)	9.9 (3.5)	-0.06	0.53	-0.12	.91	0.38	0.59	0.64	.54
Blaming Others	4-20	7.3 (3.5)	6.8 (3.9)	6.6 (1.8)	7.5 (3.5)	-0.25	0.84	-0.30	.77	0.69	0.34	2.03	.06
Rumination	4-20	11.1 (3.9)	10.1 (3.9)	9.9 (2.6)	10.0 (2.4)	-0.19	0.80	-0.23	.82	-0.06	0.88	-0.07	.94
Catastrophizing	4-20	7.0 (3.0)	6.9 (2.3)	6.9 (2.8)	6.9 (2.8)	-0.63	0.66	-0.10	.93	-0.63	0.73	-0.09	.93
**Quality of Life (KIDSCREEN-27)**	**27-135**	**107.1 (16.4)**	**106.1 (16.3)**	**101.9 (14.9)**	**105.1 (15.7)**	**-4.13**	**3.79**	**-1.09**	**.29**	**-0.94**	**3.40**	**-0.28**	**.79**
Physical Well-being	5-25	17.4 (4.9)	18.0 (4.0)	16.6 (4.0)	18.3 (3.9)	-1.44	0.92	1.57	.14	0.31	0.67	0.47	.65
Psychological Well-being	7-35	26.9 (4.5)	26.8 (4.8)	25.6 (5.5)	25.9 (5.1)	-1.25	1.30	-0.96	.35	-0.94	1.30	-0.96	.35
Autonomy and Parent Relations	7-35	31.2 (3.4)	30.5 (4.0)	30.2 (3.7)	30.9 (2.6)	-0.31	0.82	-0.38	.71	0.44	0.81	0.54	.60
Peers and Social Support	4-20	15.9 (4.0)	16.2 (3.9)	15.4 (3.9)	15.4 (3.7)	-0.81	1.15	-0.71	.49	-0.81	1.05	-0.78	.45
School Environment	4-20	15.6 (3.3)	14.6 (3.2)	14.3 (2.2)	14.6 (3.3)	-0.31	0.73	-0.43	.68	0.06	0.64	0.10	.92

Method = Restricted Maximum Likelihood. Values in bold indicate the main outcome variables. PSS, Perceived Stress Scale; CERQ, Cognitive Emotion Regulation Questionnaire; *M*, Mean; *SD*, Standard Deviation; B, Estimate; *SE*, Standard Error.* *p* <.05 ** *p* <.01.

#### 3.1.2 Pre-test (t1) to follow-up (t3)

Additionally, no effects were found from pre-test to follow-up (PSS: B = 0.25; 95% CI [-1.56, 2.01], *p* = .73; CERQ-Adaptive: B = 1.44; 95% CI [-2.43, 5.30], *p* = .44; CERQ-Maladaptive: B = 1.10; 95% CI [-2.98, 5.11], *p* = .58; KIDSCREEN-27: B = -0.94; 95% CI [-8.18, 6.30], *p* = .79) (see **Table 3**). Effect sizes were trivial: PSS: *d* = 0.03; CERQ-Adaptive: *d* = 0.11; CERQ-Maladaptive: *d* = 0.07; KIDSCREEN-27: *d* = -0.06.

#### 3.1.3 Exploratory analyses

Exploratory linear mixed-models of the subscales of the CERQ and KIDSCREEN-27 showed that participants reported a heightened amount of positive reappraising at post-test (t2) compared to pre-test (t1) (B = 1.19; 95% CI [0.25, 2.13], *p* = .02). This effect was no longer significant after correcting for multiple testing. No other significant effects were found. All results are described in more detail in **Table 3** below.

## 4 Discussion

The purpose of this Single Case Experimental Design-study was to investigate the effects of a free and autonomously usable mHealth application, *Stress Autism Mate-Junior (SAM Junior)*, on perceived stress, adaptive and maladaptive coping styles and QoL in adolescents with autism. Contrary to expectations, no significant effects of the SAM Junior app were found.

### 4.1 Perceived stress

As we did not document participants’ user experiences in this study, we can only speculate on the lack of effects of the SAM Junior app. First, the app may have failed to reduce stress levels because it was too complex for the study population. The use of mHealth requires several executive functions, such as initiating use and maintaining attention, as well as a certain amount of self-understanding (43). These abilities are generally less well developed in autistic adolescents (44, 45). Compromised executive functioning and a heightened rigidity in adolescents with autism may also have caused difficulty in applying the stress tips (46, 47), especially considering the relatively short time span of four weeks in which the app was used. Executive function problems and cognitive inflexibility may also have caused the high amount of dropout during the intervention phase. As executive functions still develop in early adulthood, (44), it may explain why autistic adults in previous studies (30, 31) were able to benefit from the SAM app, while the SAM Junior app showed no effects in autistic adolescents.

Alternatively, the use of SAM Junior may have caused extra stress in users, compromising its potential effect (48). Technostress caused by mHealth has been widely documented (49). Having to fill in a questionnaire at several fixed moments per day may have caused users to change their daily schedule, compromising their autonomy (50). For example, as mobile phones are often banned from classrooms in the Netherlands, participants may have felt compelled to use their limited break time to complete questionnaires. This indicates that the app may not have been tailored well enough to their daily schedules. Furthermore, participants’ heightened stress scores could have reminded them of their low well-being, causing more stress as a result (51, 52).

Finally, the SAM Junior app may have been ineffective due to the study population of clinical outpatients. These participants may have had higher support needs and extra stress caused by common co-occurring conditions such as anxiety disorders and post-traumatic stress disorder (53), meaning aid in managing general daily stressors was insufficient. Moreover, crisis prevention plans, that are recommended in Dutch clinical autism practice (54), serve a similar function as the SAM Junior app, potentially making the app redundant. That said, the SAM app for adults was effective in a clinical population (30, 31), meaning this alone does not fully explain the null results.

### 4.2 Coping styles

There were no changes observed in maladaptive and adaptive coping styles after use of the SAM Junior app. The stress tips of the SAM Junior were predominantly focused on behavioral change, while the CERQ measures cognitive coping strategies. This may explain the lack of change in CERQ scores. Regardless, some stress tips, such as *do a breathing exercise*, have previously shown to alter both adaptive and maladaptive cognitive coping styles in autistic adults (55). Therefore, the lack of effects of SAM Junior on coping styles suggests that some useful stress tips may not have been chosen as often or were not used as intended.

### 4.3 Quality of life

Although not statistically significant, the average QoL in participants dropped during the intervention phase. Regression to the mean may be responsible for this pattern (56). Indeed, average quality of life at baseline was higher than is commonly found in adolescents with autism (20, 57–59) and also higher compared to a cohort of Dutch allistic adolescents (60). Furthermore, it has to be noted that a change in QoL was less plausible than a change in the other variables, as perceived stress is only partially linked to QoL (61–63). This also explains why the perceived stress levels and QoL of the participants could be high at the same time.

### 4.4 Strengths and limitations

This study has several strengths. This is the first quantitative study that evaluated an mHealth application that aims to reduce stress in adolescents with autism. The app was created in co-creation with autistic adolescents which assured that the app was tailored to their needs. The questionnaires were conducted during a meeting with a researcher, which enabled the participants to ask the researcher about any unclear questions.

Several limitations also need to be mentioned. First, partially due to drop-out, this study was underpowered. This means we were not able to detect potential smaller effects. As the SAM Junior app is free and available to anyone with a smartphone, even small benefits to the well-being of autistic adolescents would have been a relevant finding. Second, although we aimed to gather data strictly at four-week intervals, some participants took longer to respond, often due to poor mental health or scheduling conflicts. This may have changed outcomes, as participants might have only completed the questionnaires during lower stress periods or may have used the app for longer than intended. Third, the adherence of the participants was not checked, meaning it is unsure to what extent the app was actually used. Therefore, a potential lack of use of the SAM Junior app may also explain the null results of this study. Fourth, due to the small sample size and the fact that all participants received assistance from a mental health care facility, as well as the exclusion of autistic adolescents with intellectual disabilities, the study sample may not have been representative of the all adolescents with autism.

### 4.5 Future research

Qualitative research about the user experience of the SAM Junior app may help to gain a better understanding on why the app was not found to be effective. We are currently conducting such a study. Based on its outcomes, the app can be improved. Additionally, recommendations from existing literature can also guide enhancements. Here, we will provide three examples. First, implementing text-to-speech options could better address the unique sensory needs of adolescents with autism (64). Second, adding gamification features (e.g. rewards for lowered stress levels) may increase user engagement and motivate users to apply stress tips in daily life (65). Third, allowing users to complete the in-app questionnaire at their preferred times, instead of at set intervals, may reduce the stress associated with using the app (50).

Once the SAM Junior app has been upgraded, the new version can be tested in a quantitative study. To increase the certainty that potential effects are caused by the use of the app, a SCED with an increased number of phases (i.e. an ABAB-design) is recommended. Alternatively, to increase the validity of the outcomes further, a randomized controlled trial can be conducted. In addition, collecting data at more time points would allow for mediation analyses. These can help to discover the exact mechanisms through which the SAM Junior app may be effective (66). Regardless of the research design, we recommend studying a non-clinical sample of autistic adolescents to prevent potential confounding effects of concurrent treatment on the study outcomes.

### 4.6 Conclusion

In sum, the results of this study do not show effectiveness of the SAM Junior app in adolescents with autism. This implies that the app may not currently be a viable alternative for reducing stress in this population. However, the limitations of this pilot study warrant caution in interpreting its outcomes. Further research with an improved application is recommended to determine the potential effects of the SAM Junior app with more certainty.

## Data Availability

The raw data supporting the conclusions of this article will be made available by the authors, without undue reservation.

## Footnotes

1 We attempted to follow Bottema-Beutel et al. (1) guidelines for avoiding ableist language in autism research. Therefore, we refrained from using the official term ‘autism spectrum disorder’.

2 Some adults with autism prefer identity-first language (‘autistic adult’) and others prefer person-first language (‘adult with autism’) (4). Therefore, in this article, these terms are used interchangeably.
